# Valorization of Tomato Stem Waste: Biochar as a Filler in Three-Dimensional Printed PLA Composites

**DOI:** 10.3390/polym17192565

**Published:** 2025-09-23

**Authors:** Dimitrios Gkiliopoulos, Sotirios Pemas, Stylianos Torofias, Konstantinos Triantafyllidis, Dimitrios N. Bikiaris, Zoi Terzopoulou, Eleftheria Maria Pechlivani

**Affiliations:** 1 Centre for Research and Technology Hellas, Information Technologies Institute, 6th km Charilaou-Thermi Road, 57001 Thessaloniki, Greece; dgiliopo@chem.auth.gr (D.G.); sopemas@iti.gr (S.P.); 2 Laboratory of Chemical and Environmental Technology, Department of Chemistry, Aristotle University of Thessaloniki, 54124 Thessaloniki, Greece; 3 Interdisciplinary Research Center for Refining and Advanced Chemicals, King Fahd University of Petroleum and Minerals, Dhahran 31261, Saudi Arabia; k.triantafyllidis@kfupm.edu.sa; 4 Chemistry Department, King Fahd University of Petroleum and Minerals, Dhahran 31261, Saudi Arabia; 5 Laboratory of Chemistry and Technology of Polymers and Colors, Department of Chemistry, Aristotle University of Thessaloniki, 54124 Thessaloniki, Greece; dbic@chem.auth.gr; 6 Laboratory of Industrial Chemistry, Department of Chemistry, University of Ioannina, 45110 Ioannina, Greece

**Keywords:** agricultural waste, pyrolysis, biochar, poly(lactic acid), 3D printing, additive manufacturing, fused deposition modeling (FDM), composite filament, biobased composites, circular economy

## Abstract

This study explores the valorization of tomato stem waste by converting it into biochar through slow pyrolysis and incorporating it into poly(lactic acid) (PLA) composites for fused filament fabrication (FFF) 3D printing. The objective was to improve the valorization and added value of tomato stem waste. Biochar derived from tomato stems was characterized for its physicochemical properties, revealing a high surface area and small particle size. PLA-based composite filaments with 5% and 7.5% biochar were manufactured via melt extrusion. The effects of biochar concentration and printing infill patterns (concentric and rectilinear) on the mechanical and thermomechanical properties of the 3D-printed composites were investigated. Results indicated that biochar slightly increased the glass transition temperature of PLA and improved the flexural properties. Dynamic mechanical analysis (DMA) showed that the storage modulus was enhanced in the glassy region for composites with 5% biochar, suggesting improved stiffness. This research demonstrates the potential of using tomato stem-derived biochar as a sustainable filler in PLA composites, contributing to the circular economy and reducing environmental impact.

## 1. Introduction

Sustainable manufacturing has emerged as a key component of industrial practices over the last years, driven by increasing environmental awareness and promising a more eco-conscious economy. Innovative strategies are developed in both academic and industrial sectors, to reduce carbon footprints, minimize waste, and enhance resource efficiency. Sustainable manufacturing practices, such as the adoption of renewable energy sources, the implementation of circular-economy principles, and the use of eco-friendly materials, are becoming integral to modern industrial operations. These efforts not only contribute to environmental preservation but also offer economic benefits by reducing operational costs and meeting the rising consumer demand for green products [1,2,3,4].

Biochar is a renewable material produced by biomass pyrolysis under zero- or very low-oxygen conditions and can contribute significantly to the implementation of sustainable manufacturing practices due to its potential environmental benefits, including carbon sequestration, soil enhancement, and waste management [5,6]. Biochar is a stable, carbonaceous substance with numerous beneficial properties, including high thermal stability, large surface area, excellent chemical stability, and electrical conductivity [7] and is often considered a greener alternative to carbon black. These properties are affected both by the biomass source and the processing conditions, and make biochar an adaptable material that can be utilized in a wide range of applications, such as agricultural and animal husbandry [8,9], catalysis [10], construction materials [11], heat and electricity generation [12,13], metallurgical applications [14], and polymer composites [15,16,17,18,19]. Biochar from various biomass sources has already been used as a reinforcing agent in different types of polymers, providing promising results. Biochar-reinforced 3D-printed polymer composites have demonstrated significantly improved mechanical, thermal, and processing properties, with enhancements attributed to both high filler loadings and surface modifications that promote interfacial compatibility [20,21,22,23,24].

Additive manufacturing (AM) or 3D printing is a transformative process that creates objects by building them layer-by-layer from digital models. Unlike traditional subtractive manufacturing, which removes material to shape an object, AM adds material precisely where needed, allowing intricate designs and complex geometries that were previously unattainable [25,26,27]. The wide range of materials utilized in additive manufacturing, including polymers, metals, ceramics, and biomaterials, significantly boosts its capability to transform manufacturing processes and product development [28,29,30,31,32,33]. In the context of polymer composites, AM is revolutionizing their production by enabling the creation of lightweight, geometrically complex parts with tailored properties via continuous processes. Techniques like Fused Deposition Modeling (FDM) and Selective Laser Sintering (SLS) are particularly prominent in this field, offering scalable solutions for developing high-performance composite structures [28,34,35,36]. The most used polymer in FDM is PLA [35].

Previously, we studied biochar as an additive in in situ prepared PLA and poly (butylene succinate) (PBS) composites [37,38,39,40,41]. We demonstrated that incorporating biochar led to well-dispersed biocomposites with improved mechanical strength, thermal properties, and controlled crystallization behavior, particularly at low filler loadings. Strong polymer–biochar interactions were shown to increase the glass transition temperature, slightly suppress crystallizability in PLA, and act as a nucleating agent in PBS, while also enhancing UV resistance and modifying degradation pathways. In another work, we demonstrated the potential of tomato stem powder (TSP) as a sustainable filler in PLA composites for FFF, showing good printability, maintaining tensile properties at 5 wt.%, and antioxidant activity, despite reduced flexural strength [42].

In this work, to improve the valorization potential of TSP, it was first converted to biochar via pyrolysis to improve filler dispersion, interfacial compatibility, and overall composite performance through its higher surface area and smaller particle size. Using biochar derived from tomato stem waste offers a sustainable and innovative way to valorize an abundant, nonedible agricultural residue, reducing environmental impact and supporting circular economy practices. Compared to other biomass sources, tomato stems yield biochar with tailored surface chemistry and moderate porosity, potentially enhancing compatibility with biopolymers like PLA and improving composite performance [43]. Composite PLA-based filaments with biochar were manufactured via melt extrusion. The effects of biochar concentration and the printing infill patterns (concentric and rectilinear) on the mechanical and thermomechanical properties of 3D-printed PLA composites were studied.

## 2. Materials and Methods

### 2.1. Materials

Greenhouse tomato stems (*Solanum lycopersicum* L.) were used as the biomass source for biochar production. The tomatoes were cultivated in 2024 at the greenhouse demonstration sites of the EU project E-SPFdigit (Grant Agreement ID: 101157922), funded by Horizon Europe. 4043D type PLA was supplied by 3devo (Utrecht, The Netherlands) in pellet form and was used as the polymer matrix. Joncryl ADR^®^ 4400, a multifunctional polymer with an epoxy equivalent weight of 485 g/mol and a weight-average molecular weight of 7100 g/mol, was used as the chain extender and was provided by BASF (Ludwigshafen, Germany).

### 2.2. Production of Biochar from Tomato Stem Waste

#### 2.2.1. Grinding of Tomato Stem Waste

Tomato plant pruning was processed into a fine powder by applying alternate stages of segmentation and drying. First, tomato stems were chopped into small pieces and then dried in a vacuum oven at 80 °C overnight. Once dried, they were milled using a knife mill (Moulinex LM811 household blender, 1200 W, Groupe SEB, Ecully, France) at half speed (approximately 14,000 rpm) for 30 min. The resulting fibrous particles were then subjected to a second vacuum drying at 80 °C to ensure complete moisture removal, followed by a second round of milling under the same conditions, giving a finely powdered product.

#### 2.2.2. Biochar Production via Slow Pyrolysis of Tomato Stem Powder

The slow pyrolysis of tomato stem powder was conducted in a custom-made, bench-scale, downflow, fixed-bed, tubular reactor, made of stainless steel 316. A total of 50 g of pre-dried tomato stem powder with a particle diameter of 250–1000 μm was inserted in the reactor, which was heated externally by an electric oven with a heating rate of 20 °C/min. The carbonization reaction took place at 500 °C, for 4 h under a nitrogen atmosphere. The resulting biochar was placed in a desiccator until it reached room temperature, and the reaction yield was calculated using Formula (1).

(1) $$yield\;\%=\frac{biochar\;mass}{feeded\;biomass\;mass}\times 100$$

### 2.3. Production of PLA/Biochar Composite Filaments

For the present study, two types of filaments incorporating biochar and one type of neat PLA filament, used as a reference, were developed. The objective was to utilize as much biochar as possible. Several filaments with varying proportions of biochar were tested, but with the materials and methods used in this study, filaments containing more than 7.5% (wt.%) biochar were not suitable for FFF 3D printing. The two filaments that were successfully created contained 5% and 7.5% biochar, and were referred to as PLA-5% Biochar and PLA-7.5% Biochar, respectively. Both filaments also included 2% Joncryl. Specifically, the PLA-5% Biochar filament consisted of 93% (wt.%) PLA, 2% (wt.%) Joncryl, and 5% (wt.%) biochar, while the PLA-7.5% Biochar filament consisted of 90.5% (wt.%) PLA, 2% (wt.%) Joncryl, and 7.5% (wt.%) biochar. For the development process, PLA pellets were first vacuum-dried overnight at 40 °C, then mixed with biochar and Joncryl, which improves the physicochemical properties of the resulting filaments [44]. The 3D printing filaments were produced using the 3devo Composer Series 350/450 filament maker (Utrecht, The Netherlands). The diameter of the final filaments fluctuated within a range of ±0.07 mm. Due to the formation of biochar particle agglomerates, the filament diameters ranged from 1.68 mm to 1.82 mm at a few specific points. However, no extrusion issues were observed during the 3D printing process. The filament maker uses four heating zones to melt and homogenize the materials (PLA, Biochar, Joncryl) before extruding them into a composite FFF filament via a mixing screw, which was set at 3.2 rpm for this study. The temperatures of the four heating zones were as follows: Heater 1 (H1) at 175 °C, Heater 2 (H2) at 180 °C, Heater 3 (H3) at 180 °C, and Heater 4 (H4) at 175 °C. Heater 4 is the first zone the material passes through, followed by Heater 3, Heater 2, and finally Heater 1, which extrudes the material through a nozzle. After extrusion, the melted material was immediately cooled using two fans set at 90% of their maximum power. Table 1 summarizes the developed filaments and their respective compositions.

### 2.4. Additive Manufacturing Process (Specimens with Rectilinear and Concentric Patterns)

**Figure 1:**
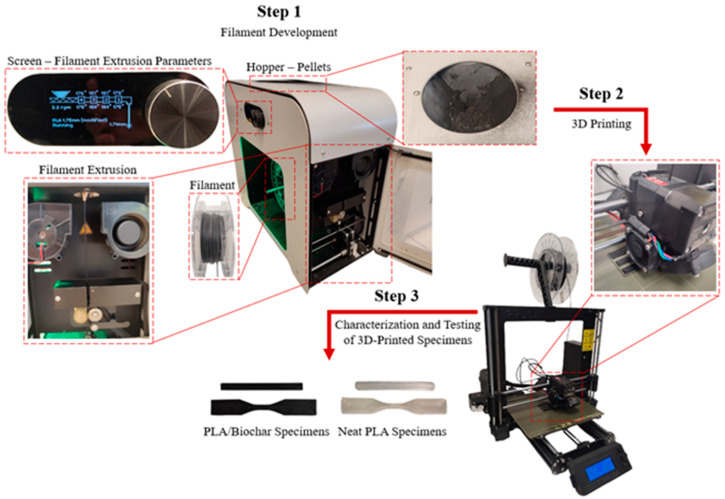
Illustration of the entire additive manufacturing process.

To investigate the properties of the developed neat PLA and PLA/biochar composite filaments, two different types of specimens were designed and printed. For the tensile test, the specimens were sized according to the international standard ASTM D638 [45], Type V. For flexural strength and Dynamic Mechanical Analysis (DMA), specimens were sized at 50 × 5 × 2 mm. The mechanical designs of the test specimens were created using SOLIDWORKS^®^ CAD Software (2022 SP2.0 Professional version), and the STL files were then inserted into Prusa Slicer 2.5.2 to adjust printing parameters and export the G-code for the Original Prusa i3 MK3S+ FFF 3D printer (Prusa Research a.s., Prague, Czech Republic). From the three developed filaments (Neat PLA, PLA-5% Biochar, and PLA-7.5% Biochar), specimens were printed with two different infill patterns, concentric and rectilinear, both with a 100% fill density. These infill patterns were selected to gain better insights into mechanical properties, load distribution, and stress scenarios, and to identify the most suitable pattern for future applications [46,47]. Apart from the infill pattern, all other printing parameters were kept the same across all specimens to ensure consistency. The nozzle temperature was set at 215 °C and the bed temperature at 60 °C, like standard commercial PLA settings. To ensure proper extrudability and avoid issues with potential biochar powder agglomerates, a 0.8 mm nozzle and 0.3 mm layer height were used. Details of the printed specimens, including their characteristics and quantity, are presented in Table 2, while Figure 1 illustrates the specimens and the equipment used.

### 2.5. Characterization and Testing

#### 2.5.1. Elemental Analysis

The elemental composition (C/H/N/S) of the biochar sample was determined using an EA3100 Series CHNS-O Elemental Analyzer (Eurovector, Pavia, Italy). Sample analysis was conducted in combustion mode at 980 °C with a constant helium (He) flow. Calibration was performed prior to analysis using the standard compound 2,5-bis(5-tert-butyl-2-benzo-oxazol-2-yl)thiophene (BBOT). Oxygen content was determined by difference.

#### 2.5.2. Dynamic Light Scattering (DLS)

Particle size distribution (PSD) of biochar was determined using dynamic light scattering on a Litesizer 500 instrument (Anton Paar, Graz, Austria). Biochar was dispersed in water at a concentration of 100 ppm via ultrasonication for 5 min before measurement.

#### 2.5.3. N2 Physisorption

The N_2_ porosimetry measurement of biochar was conducted by obtaining the adsorption/desorption isotherms at −196 °C on an Automatic Volumetric Sorption Analyzer (Autosorb-1MP, Quantachrome Instruments, Boynton Beach, FL, USA). The powder was outgassed at 130 °C and 1.33 × 10^−4^ Pa for a minimum of 18 h prior to analysis. The specific surface area (S_BET_) was estimated by the multipoint Brunauer–Emmett–Teller (BET) method using the adsorption isotherm points in the range of 0.05 < P/P_0_ < 0.20, and the total pore volume (V_p_) was determined by the adsorbed nitrogen at P/P_0_ = 0.99.

#### 2.5.4. Thermal Gravimetric Analysis (TGA)

The thermal stability of biochar was determined via TGA, which was conducted using a Netzsch STA 449F5 instrument (Netzsch Group, Selb, Germany). Biochar was placed in an alumina crucible and was heated under a 50 mL/min flow of N_2_ and a heating rate of 10 K/min in the temperature range of 25–950 °C. The same conditions were applied for the TGA of PLA/biochar composites.

#### 2.5.5. FTIR Spectroscopy

ATR spectra of the samples were recorded using an IRTracer-100 (Shimadzu, Kyoto, Japan) equipped with a QATR™ 10 Single-Reflection ATR Accessory with a Diamond Crystal. The spectra were collected in the range from 450 to 4000 cm^−1^ at a resolution of 2 cm^−1^ (a total of 16 co-added scans), while the baseline was corrected and converted into absorbance mode.

#### 2.5.6. Scanning Electron Microscopy (SEM)

The examination of the surface of the PLA/biochar composite samples was conducted using a JEOL JMS 7610 F scanning microscope (Freising, Germany). Carbon coating was used to render the samples electrically conductive. The operating conditions were the following: accelerating voltage 10 kV, probe current 45 nA, and counting time 60 s.

#### 2.5.7. Differential Scanning Calorimetry (DSC)

Differential Scanning Calorimetry (DSC) analysis was performed using a Perkin Elmer Pyris 2 DSC (Shelton, CT, USA) differential scanning calorimeter, calibrated with pure indium and zinc standards. Samples of 5 ± 0.1 mg sealed in aluminum pans were used to test the thermal behavior of the quenched polymers. The degree of crystallinity was calculated using the following equation:

(2) $$Xc\left(\%\right)=\frac{\Delta Hm-\Delta Hcc}{{\Delta H}_{m}^{0}*(1-\frac{biochar\;wt\%}{100})}*100$$

where *X_c_* is the % crystallinity, ΔH_m_ is the melting enthalpy, ΔH_cc_ is the cold crystallization enthalpy, and ${\Delta H}_{m}^{0}$ is the melting enthalpy of 100% crystalline PLA (93 J/g).

#### 2.5.8. Mechanical Properties

The mechanical response of the materials during tensile and three-point flexural tests was evaluated using an Instron 3344 dynamometer (Instron, Norwood, MA, USA) equipped with a 2 kN load cell at a crosshead speed of 5 mm/min.

For the tensile tests, 3D-printed dumbbell-shaped tensile type V test specimens with dimensions 24 × 3.5 × 3.5 mm were employed. Five specimens were tested for each sample, and the resulting data were averaged to derive the mean values for Young’s modulus, stress at break, and elongation at break.

For the three-point flexural tests, rectangular specimens with dimensions 50 × 5 × 2 mm were employed. Five specimens were tested for each sample, using a 20 mm long holder, and the resulting data were averaged to derive the mean values for modulus of elasticity, flexural strength, and flexural strain at break.

Two-way ANOVA was used to determine the statistical significance. The software used was IBΜ SPSS 29.

#### 2.5.9. Dynamic Mechanical Analysis (DMA)

Dynamic mechanical analysis was used to measure the thermomechanical properties of the 3D-printed composites using a Perkin Elmer Diamond DMA analyzer (PerkinElmer Inc., Waltham, MA, USA). The bending method was applied with an oscillation frequency of 1 Hz and a heating rate of 4 °C/min. The specimens were tested from 25 to 80 °C with a 10 mN applied force. A 20 mm long holder was used. Storage modulus (E’) and glass transition temperature (Tg) were determined for each sample.

## 3. Results

In the present study, various physicochemical and mechanical characterizations were conducted to investigate and evaluate the behavior of the developed composites with biochar. The outcome of the procedures and tests conducted during the physicochemical characterization of biochar revealed a significant influence on the performance of the developed PLA composites. Elemental analysis confirmed the high carbon content, while particle size distribution and porosity measurements indicated a favorable structure for interfacial bonding. FTIR spectra demonstrated the presence of oxygenated functional groups that enhance compatibility with the PLA matrix, and TGA/DTG analyses showed the improved thermal stability of biochar. Furthermore, SEM images revealed better dispersion and stronger interfacial adhesion, validating the enhanced mechanical and thermomechanical performance of the composites.

### 3.1. Biochar Characterization

Physicochemical characterizations were performed to provide basic information on the biochar produced prior to its use as an additive of 3D-printed PLA composites, and the results are listed in Table 3 and Table 4. The method applied in the slow pyrolysis of tomato stem waste produced biochar with a yield of 46%.

According to the elemental analysis results, the content of the produced biochar is C: 61.93%, H: 2.38%, N: 2.21%, and O: 33.48%. These values are consistent with the results of other studies on tomato-derived biochars via pyrolysis at similar conditions [48,49].

**Figure 2:**
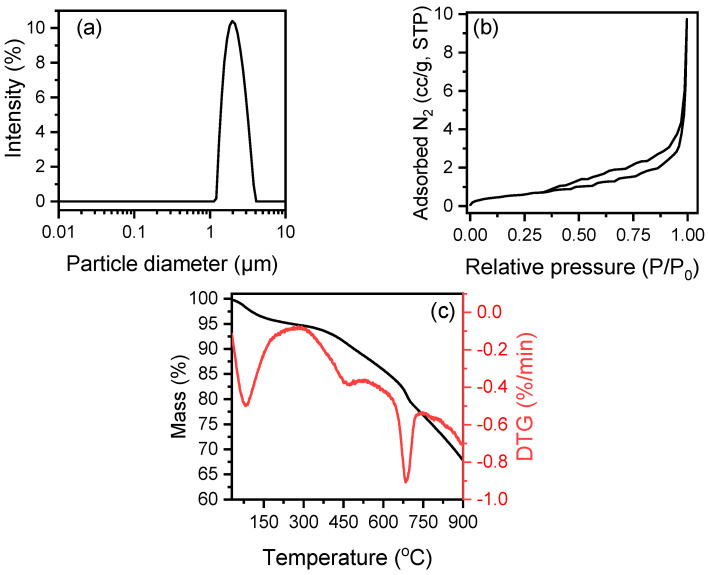
Biochar characterization: (**a**) particle size distribution, (**b**) N2 adsorption−desorption isotherm, and (**c**) TGA and DTG curves.

DLS analysis of the biochar resulted in a single-peaked PSD curve with narrow distribution and a maximum point at 2.2 μm (Figure 2a). Furthermore, the hydrodynamic diameter of the biochar particles was measured to be equal to 2.0 μm. The great reduction in biomass particle size during the pyrolysis process, known as biomass shrinkage, is due to bulk volume (mainly volatiles) loss and porosity gain, and it is well documented in the bibliography [50,51].

The N_2_ adsorption–desorption isotherm of the produced biochar is given in Figure 2b. The biochar exhibits a Type II isotherm according to IUPAC classification [52], which is the normal form of isotherm obtained with macroporous or non-porous materials. The BET analysis resulted in a specific surface area, S_BET_, of 2.048 m^2^/g, while the pore volume, V_p_, at relative pressure P/P_0_ = 0.99 was 0.015 cc/g. Although the porosity of biochar increases for carbonization processes with medium to high temperatures (500–900 °C), it is possible for the results of N_2_ porosimetry may indicate small pores and specific areas. This may be due to the blocking of the pores by inorganic components (ashes) or their structural breakdown due to heating at high temperatures for long periods of time [53].

Regarding the thermal gravimetric analysis, the TG and DTG curves of the biochar are shown in Figure 2c. The TG curve consists of three stages. The first stage, ranging from 24 to 220 °C and corresponding to a weight loss of 4.65%, is due to the evaporation of moisture and other volatile components of the sample. The second stage, ranging from 220 to 520 °C and corresponding to a weight loss of 9.18%, indicates the thermal degradation of lignocellulosic constituents. The final stage, which corresponds to gradual weight loss at temperatures higher than 520 °C, can be attributed to the thermal decomposition of inorganic components or minerals with low melting/calcination points, and to further condensation of partially pre-condensed lignocellulosic matter.

### 3.2. PLA/Biochar Composites Properties

#### 3.2.1. Morphological and Structural Characterization

**Figure 3:**
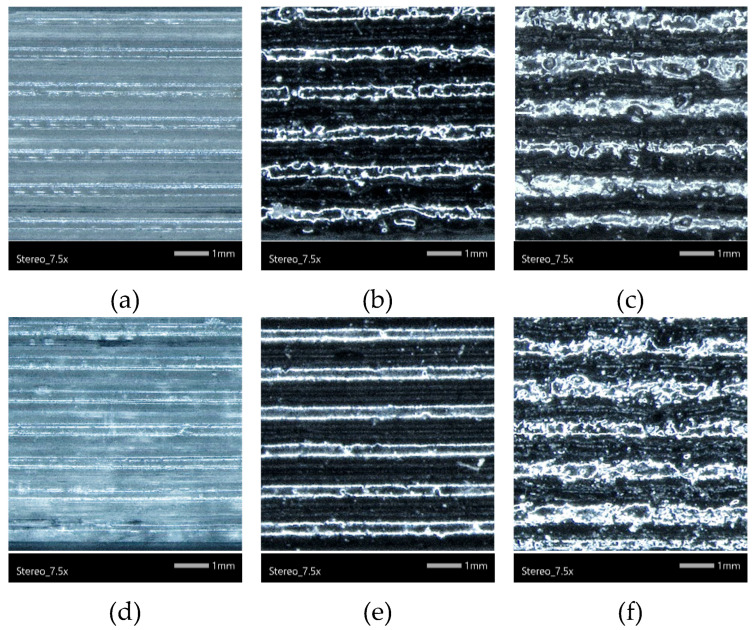
Stereoscope images of side views of (**a**) Neat PLA (concentric), (**b**) 5% biochar (concentric), (**c**) 7.5% biochar (concentric), (**d**) Neat PLA (rectilinear), (**e**) 5% biochar (rectilinear), and (**f**) 7.5% biochar (rectilinear). Magnification ×7.5.

The present study investigates the impact of incorporating varying concentrations of biochar, in conjunction with different infill patterns, on the properties of 3D-printed PLA. Layer adhesion was visually examined by stereoscopic images, shown in Figure 3. The repeated layers of PLA are visible, and overall uniform printed structures were obtained, with no visible delamination. The addition of biochar did not affect the printing quality or layer adhesion, suggesting good compatibility between PLA and biochar, and biochar content was low enough not to interfere with melt bonding between the layers. The surface roughness increased due to the particulate nature of the biochar.

**Figure 4:**
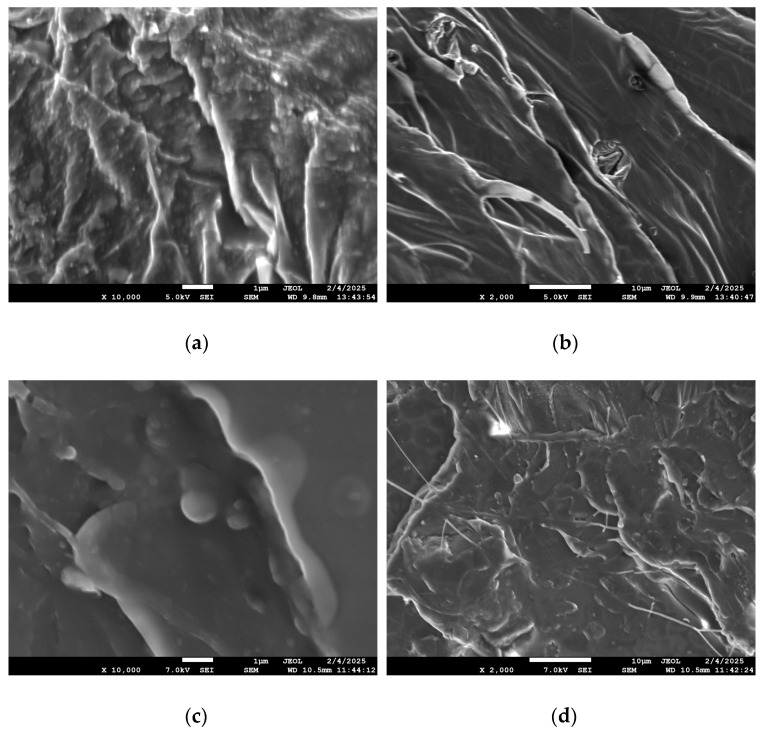
SEM micrographs of 3D-printed PLA/biochar composites. (**a**,**b**) 5% biochar, and (**c**,**d**) 7.5% biochar.

The morphology of PLA/biochar composites was also examined with SEM. According to the images taken (Figure 4), biochar was effectively dispersed inside the polymer matrix, both for 5 and 7.5% loading (Figure 4a and Figure 4c, respectively). The dispersed biochar particle size varied from the sub-micron scale (Figure 4a,c) to some aggregates (Figure 4b,d) with a size of a few micrometers, in agreement with the DLS particle size. Thus, the biochar particles were effectively dispersed in the PLA matrix during filament production. There was no evidence of phase separation. Concerning the coherence of the polymer matrix, no cracks were detected, and the sample had a continuous surface.

**Figure 5:**
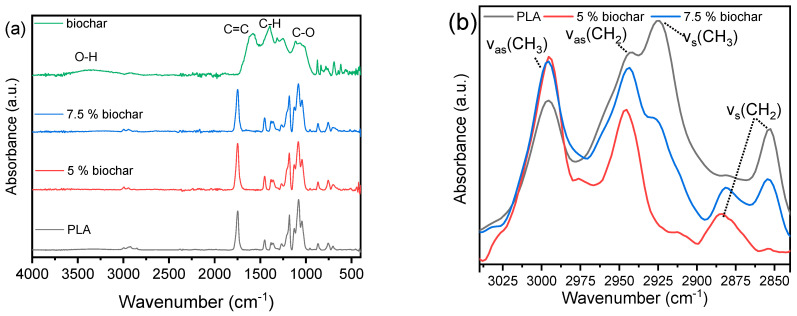
(**a**) FTIR spectra of biochar, 3D−printed PLA, and its composites with biochar, (**b**) zoom−in the range of 3040−2840 cm^−1^.

The FTIR spectrum of the produced biochar (Figure 5a, green line) exhibits characteristic absorption bands indicative of a predominantly aromatic and oxygenated carbon structure. A strong peak at 1580 cm^−1^ corresponds to the C=C stretching vibrations of aromatic rings, signifying the presence of graphitic or condensed polyaromatic domains formed during pyrolysis. The signals at 870 and 832 cm^−1^, assigned to out-of-plane C–H bending modes of substituted aromatic structures, along with the 778 cm^−1^ signal, assigned to aromatic ring deformation modes, further support the development of a condensed aromatic carbon framework. Peaks observed at 1340, 1315, and 1250 cm^−1^ are attributed to C–O–C or O–H vibrations, suggesting the presence of oxygenated functionalities such as phenols, ethers, or carboxylic acids [54]. A broad absorption around 1100 cm^−1^ is consistent with C–O stretching, indicating partial retention of lignocellulosic components or their thermally modified derivatives, and it could overlap with Si–O–Si vibrations from mineral ash. These spectral features align with the elemental composition of the biochar (Table 3).

PLA had its characteristic absorption bands at 3000–2900 cm^−1^ (C–H stretching), at 1747 cm^−1^ (C=O stretching), 1452 cm^−1^ (–CH_3_ bending), 1180 cm^−1^ (C–C–O stretching), and 1082 cm^−1^ (O–C–C stretching). Upon incorporation of biochar (Figure 5b), a new band appeared at 2880 cm^−1^, attributed to a shifted or additional symmetric CH_2_ stretching mode, likely arising from altered interfacial environments or chain conformations. Simultaneously, the intensity of the 2852 cm^−1^ peak decreased, suggesting disruption of native PLA chain packing, which could be related to decreased chain mobility. The 2943 cm^−1^ band became more intense, while the 2924 cm^−1^ band decreased, pointing to selective interactions between PLA aliphatic segments and biochar surface functionalities. The increased intensity of the asymmetric CH_2_ stretch at 2943 cm^−1^ implies that methylene groups become more exposed or disordered, while the decrease in the symmetric CH_3_ stretch at 2924 cm^−1^ indicates restricted mobility of methyl groups, possibly caused by steric hindrance or local packing effects at the biochar interface. The lack of shifts in the carbonyl region (1747 cm^−1^) implies the absence of strong covalent bonding, pointing to physical interactions at the PLA–biochar interface [55].

#### 3.2.2. Thermal Transitions

**Figure 6:**
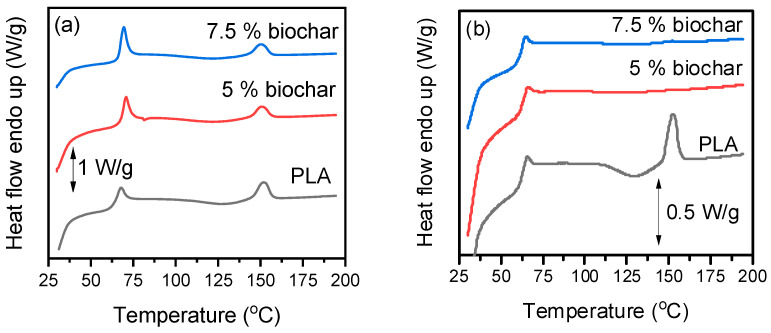
DSC scans of 3D-printed PLA and its composites with biochar: (**a**) 1st heating and (**b**) 2nd heating.

To evaluate how biochar affects the thermal transitions of PLA, DSC scans were conducted. Figure 6 shows the results from the first heating (as-printed samples) and the second heating. The corresponding values for glass transition temperature (Τ_g_), cold crystallization temperature (T_cc_), melting temperature (T_m_), and crystallinity (*X_c_*) are listed in Table 5. The first heating scan (Figure 6a) reflects the thermal history from the 3D printing process, while the second heating scan (Figure 6b) reveals the inherent thermal behavior of the composites.

The addition of biochar slightly increased the Τ_g_ during the first heating scan, indicating restricted chain mobility due to the physical interactions between PLA and the filler. This increase is weaker in comparison to PLA/biochar composites prepared by in situ polymerization, as in the latter case, strong covalent interactions occur between the PLA and biochar [39]. In the second heating scan, only the 5% biochar composite had a slightly lower increase in T_g_, which could be a result of the relaxation of residual stresses and the removal of physical aging effects during the initial heating cycle. The T_g_ of 7.5% biochar remained the same, which could be caused by less well-dispersed biochar, leading to less uniform polymer filler interactions and thus minimizing its effect in restricting chain mobility, or a reduction in the polymers’ molecular weight during thermal processing [56]. There was no evidence of increased –COOH or –OH groups in the FTIR spectra (Figure 5) or visible degradation in the SEM images (Figure 4), but it cannot be excluded, as biochar contents >5 wt% can accelerate the thermal degradation of PLA [39]. To minimize thermal degradation, the biochar used in this study was thoroughly dried and selected for its fully carbonized structure, which ensured a low content of surface functional groups (e.g., –OH, –COOH) and the absence of mineral residues. These characteristics reduce the risk of catalytic degradation of PLA during processing.

The T_cc_ decreased with increasing biochar content in both scans, indicating a possible nucleating effect of biochar that promotes crystallization at lower temperatures [57,58], but it was still very limited and occurred only during heating. This is associated with the inherent weak crystallizability of PLA as well as the high molecular weight of the PLA grade used. Finally, the T_m_ showed a minor decrease across compositions (from 152 °C to ~150 °C), likely due to less perfect crystals forming in the presence of biochar.

#### 3.2.3. Mechanical and Thermomechanical Properties

**Figure 7:**
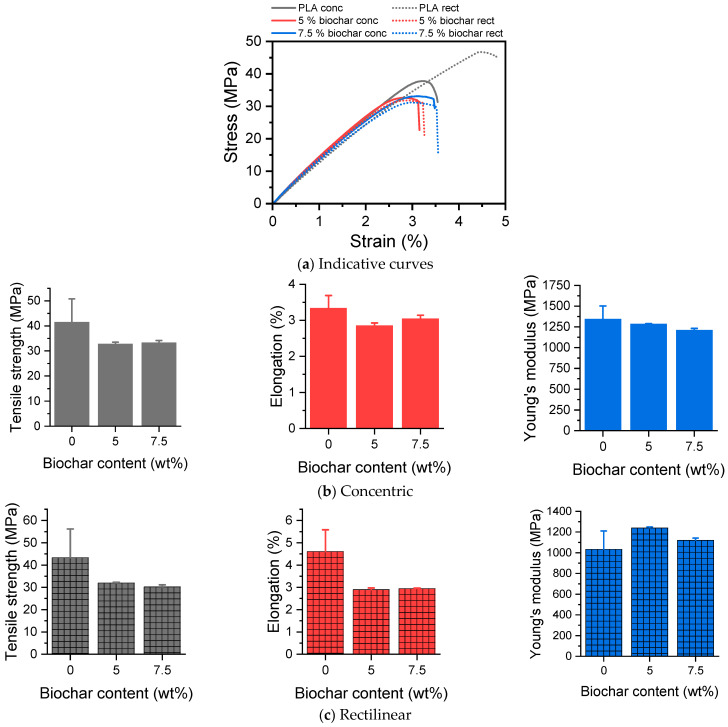
(**a**) Indicative tensile stress–strain curves, tensile properties of 3D-printed PLA/biochar composites: tensile strength σ_b_, elongation ε_b_, and Young’s modulus Ε_b_ of (**b**) concentric and (**c**) rectilinear specimens.

The mechanical properties of PLA/biochar composites, as evaluated by tensile testing, are shown in Figure 7. The stress–strain curves of the PLA/biochar composites (Figure 7a) are typical of hard and brittle materials, with an initial linear region, corresponding to elastic behavior, followed by a nonlinear region until the maximum stress point, where failure takes place. The rectilinear PLA had higher tensile strength and elongation at break (σ_b_ = 46.8 ± 12.8 MPa, ε_b_ = 4.6 ± 1.0) than concentric PLA (σ_b_ = 41.5 ± 9.2 MPa, ε_b_ = 3.3 ± 0.3). The grid-like structure of the rectilinear infill might distribute stress more evenly in multiple directions, since it has more intersections and contact points between layers [59].

The effect of biochar content on the tensile properties of PLA concentric and PLA rectilinear is presented in Figure 7b and Figure 7c, respectively. The average σ_b_ and average ε_b_ varied between 30 and 43 MPa, and between 2.9 and 4.6%, respectively, while Young’s modulus E_b_ was between 1036 and 1347 MPa. Overall, an effect was observed of the biochar on the σ_b_ (*p* = 0.025) irrespectively of the infill pattern. In more detail, the σ_b_ was significantly reduced when using 5% biochar (*p* = 0.049) and 7.5% biochar (*p* = 0.036) in comparison with neat PLA. When examining the ε_b_, an overall effect of the biochar (*p* = 0.001) was observed as well as an interaction effect (*p* = 0.031). More specifically, compared to neat PLA, the ε_b_ of both 5% biochar (*p* = 0.002) and 7.5% biochar (*p* = 0.005) was significantly reduced. In more detail, when PLA was printed with rectilinear infill, the ε_b_% of 5% biochar (*p* = 0.02) as well as of 7.5% biochar (*p* = 0.026) was significantly lower than that of neat PLA. When examining the E_b_ values, only an overall effect of the infill pattern (*p* = 0.007) was observed. This was present between the 5% biochar of the concentric infill and that of the rectilinear (*p* < 0.001) and between both 7% biochars (*p* = 0.004). The reduction in the tensile properties of PLA with biochar is common in the literature [58,60,61,62], and it is attributed mainly to aggregation. While biochar additions way below 5 wt% may improve or maintain the mechanical properties of PLA due to better dispersion and minimal aggregation [60,63,64], such low loadings may not sufficiently valorize the filler, limiting potential environmental or economic benefits. Thus, there is a critical balance between enhancing mechanical performance and incorporating enough biochar to justify its use in terms of sustainability and material value.

**Figure 8:**
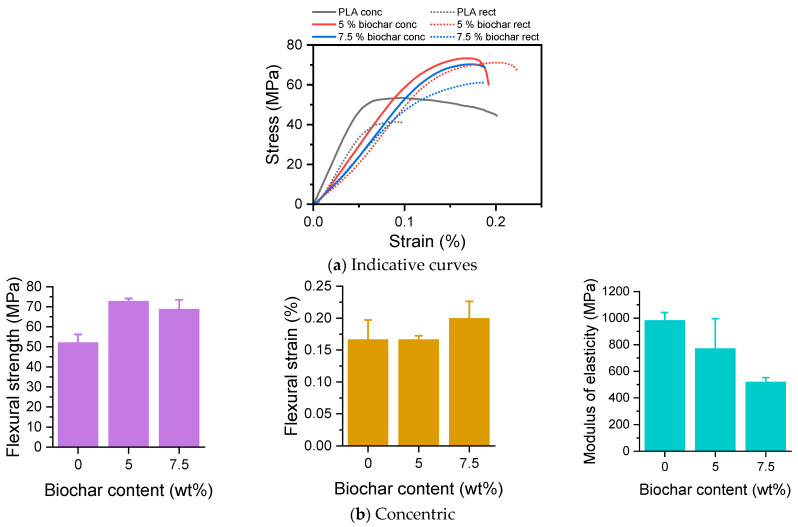

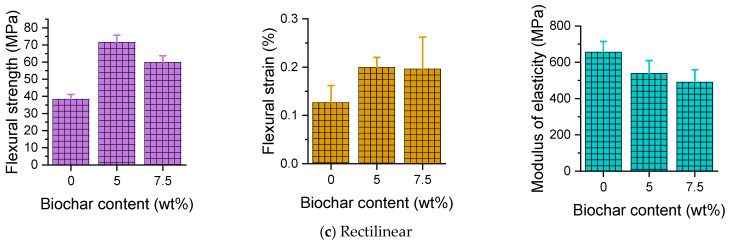
(**a**) Indicative flexural stress–strain curves, Flexural properties of 3D-printed PLA/biochar composites: flexural strength σ_f_, flexural strain ε_f_, and flexural modulus E_f_ of (**b**) concentric and (**c**) rectilinear specimens.

Figure 8 shows the response of 3D-printed PLA/biochar composites to the bending test. As illustrated in Figure 8a, the flexural stress–strain curves of the samples correspond to hard and brittle materials with similar characteristics. In contrast to the tensile tests, all samples show higher flexural strength, σ_f_, compared to the pristine polymer [42], but lower modulus of elasticity, E_f_. The effect of biochar content on the flexural properties of PLA concentric and PLA rectilinear is presented in Figure 8b,c. The average σ_f_ and average strain at break, ε_f_, varied between 38 and 73 MPa, and between 0.1 and 0.2%, respectively, while E_f_ was between 492 and 984 MPa. Overall, an effect was observed of the biochar (*p* < 0.001) as well as the infill pattern (*p* < 0.001) on the σ_f_. Furthermore, an interaction effect was present (*p* = 0.30). In particular, the σ_f_ was significantly increased when using 5% biochar (*p* < 0.001) and 7.5% biochar (*p* < 0.001) in comparison with neat PLA. When comparing 5% with 7.5% biochar, the former was significantly higher than the latter (*p* = 0.006), suggesting that moderate biochar loading improves σ_f_ more effectively than higher loading, which may introduce defects or agglomeration. Petousis et al. also found that 4–6 wt. % was the optimum loading for the mechanical properties of PP, HDPE, and PLA composites with biochar from olive tree prunings [20,21,24]. When PLA was applied concentrically, only the 5% (*p* < 0.001) and the 7.5% biochar were significantly different than neat PLA. If PLA was applied rectilinearly, significant differences were observed between neat PLA and 5% biochar (*p* < 0.001), neat PLA and 7.5% biochar (*p* < 0.001), and between 5% and 7.5% biochar (*p* = 0.015). Upon comparing concentric PLA with rectilinear PLA, only both neat PLAs differed significantly from another (*p* = 0.007). In contrast to the afore-mentioned σ_f_ differences, no differences were observed when examining the ε_f_.

Finally, the E_f_ were examined. Upon comparing, an overall effect of the infill geometry (*p* = 0.002) as well as the biochar addition (*p* < 0.001) on the E_f_ was found. In more detail, the E_f_ was significantly reduced when using 5% biochar (*p* = 0.047) and 7.5% biochar (*p* < 0.001) in comparison with neat PLA. More specifically, when PLA was printed concentrically, the 7.5% biochar was significantly lower than the neat PLA (*p* = 0.013). This was also observed if PLA was printed rectilinearly, as only the 7.5% biochar was significantly lower than neat PLA (*p* = 0.45). Upon examining any differences between concentric and rectilinear applied PLAs, only the neat PLAs differed from one another (*p* = 0.002). These results agree with the literature, where different types of biochar could also improve the flexural properties of PLA [60] due to the inherent hardness of biochar that limits the flexural deformation of the PLA matrix.

**Figure 9:**
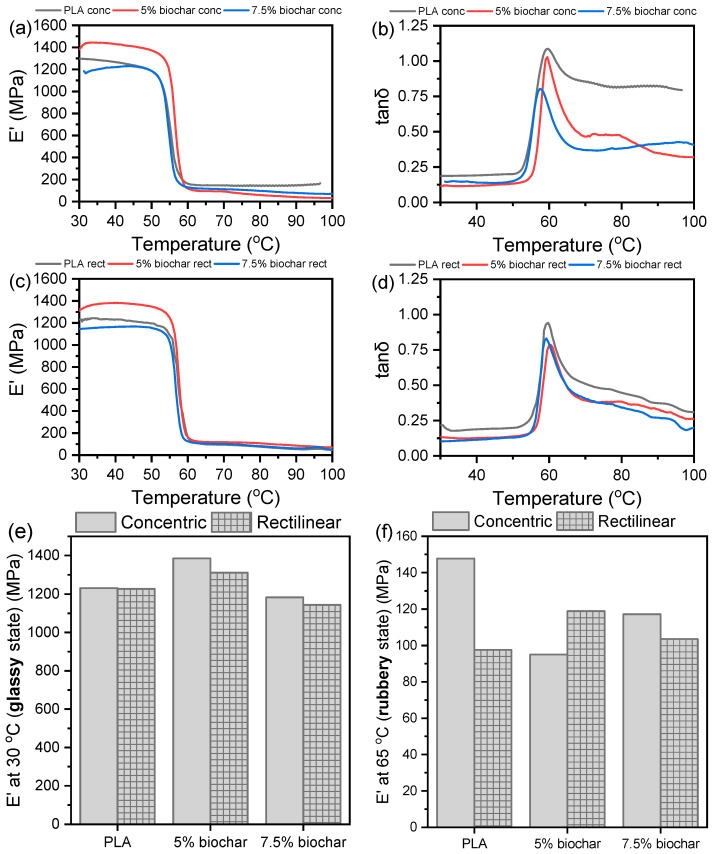

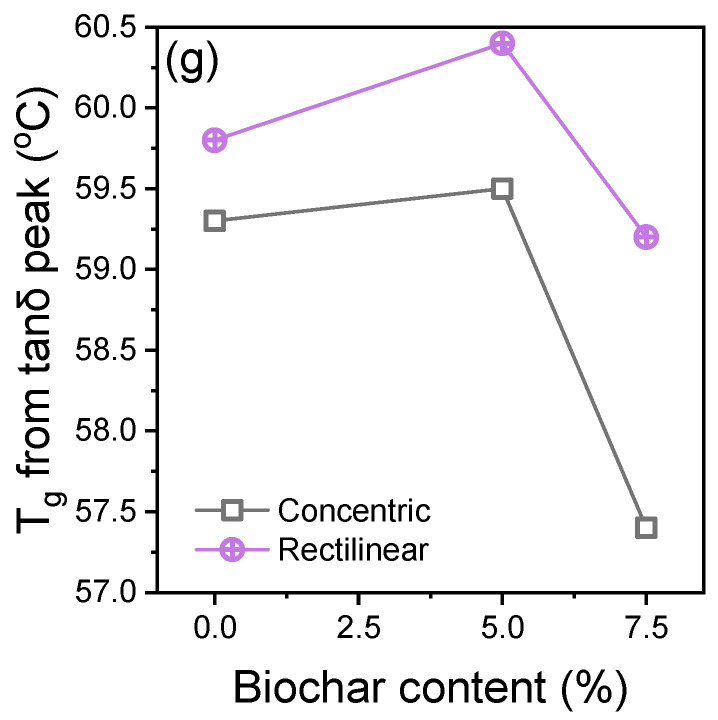
DMA of 3D-printed PLA/biochar composites, (**a**) storage modulus, E’, of concentric specimens, (**b**) tanδ of concentric specimens, (**c**) storage modulus, E’, of rectilinear specimens, (**d**) tanδ of rectilinear specimens, storage modulus values, E’, at glassy (**e**) and rubbery (**f**) glass transition temperature, T_g_, (**g**) Effect of biochar content and printing pattern on the T_g_ derived from the tanδ peak temperature.

The viscoelastic properties of PLA/biochar composites were studied with DMA, and the results are presented in Figure 9. The storage modulus E’ in the glassy region of the composite samples with 5% biochar (Figure 9e,f) was higher compared to that of the pristine polymer, especially for the concentric infill (from 1298 MPa to 1385 MPa), suggesting enhanced stiffness. This is consistent with literature [65,66], where moderate biochar loading can act as a reinforcing filler by restricting chain mobility and improving stiffness. However, a reduction in E′ at the rubbery region (65 °C) was observed for all biochar composites relative to neat PLA. This softening above T_g_ reflects weakened polymer-filler interfacial adhesion and/or early onset of thermal degradation, especially at higher temperatures. In fact, T_g_ values from tan δ peaks (Figure 9b,d,g) showed a modest increase with 5 wt% biochar, from 59.3 °C to 60.4 °C for rectilinear samples. This suggests some degree of chain immobilization due to biochar interaction. However, at 7.5 wt% biochar, T_g_ decreased to 57.4 °C (concentric) and 59.2 °C (rectilinear), indicating reduced chain cohesion, possibly due to agglomeration or chain scission. Moreover, the height and sharpness of the tan δ peaks (Figure 9b,d) remained well-defined and narrow, indicating good phase homogeneity with a T_g_, unlike in blends or partially miscible composites, where broad transitions suggest multiple relaxation processes.

The test results of all the mechanical and viscoelastic properties characterization are listed in Table 6. Interestingly, despite the drop in viscoelastic stiffness in the rubbery regime, flexural strength improved with 5% biochar, as discussed previously. This increase in strength, even as dynamic stiffness dropped at higher temperatures, suggests that biochar reinforces the composite below T_g_, while its benefits diminish near or above T_g_ due to poor thermal stability. Furthermore, the influence of the infill pattern is evident. The rectilinear specimens consistently demonstrated slightly higher T_g_, tensile strength, and rubbery-region storage modulus than concentric ones. This is consistent with the hypothesis that the grid-like interlocking structure of rectilinear infill improves stress distribution and layer bonding, enhancing both load transfer efficiency and dynamic stiffness retention near T_g_. In contrast, concentric patterns, while better at certain stress directions, may introduce anisotropy and weaker inter-path fusion, limiting their performance under complex stress.

The increase in storage modulus at 30 °C observed in PLA composites with 5 wt% biochar aligns with Wei et al. [57], who also reported modulus enhancement at moderate filler loadings due to improved particle-matrix interaction and restricted chain mobility. However, similar to both Wei et al. [57] and Kane et al. [56], higher filler content (7.5%) in our study led to a decline in E′ and T_g_, likely due to filler agglomeration or chain degradation, which weakens interfacial bonding. Moreover, the reduction in E′ at the rubbery region and drop in T_g_ is also consistent with the findings of Kane et al. [56], who attributed such behavior to biochar-induced thermal degradation and poor chain cohesion at elevated temperatures.

**Figure 10:**
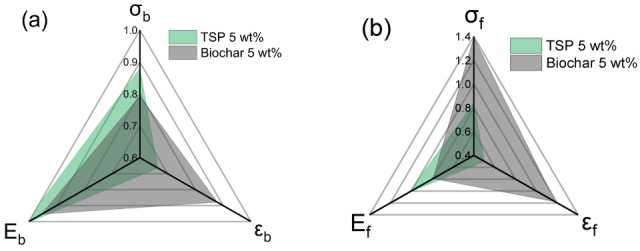
Comparison of mechanical properties of TSP [42] and biochar composites (5 wt% filler, concentric) normalized to neat PLA (neat PLA = 1). (**a**) tensile properties and (**b**) flexural properties.

Previously, we studied the use of tomato stem powder (TSP) as an additive in PLA without carbonization [42]. In Figure 10, we compare the tensile properties of concentric PLA with 5 wt% TSP and 5 wt% biochar from TSP. The results indicate opposing effects on tensile and flexural properties, highlighting the influence of biochar’s physicochemical transformation during pyrolysis. While TSP led to a modest increase in Ε_b_, it reduced σ_b_ and ε_b_, likely due to its large particle size and mediocre dispersion. In contrast, biochar (~2 μm) still reduced σ_b_ but maintained ε_b_ and significantly enhanced σ_f_ (by 39%) and ε_f_, despite a slight decrease in Ε_f_. FTIR analysis provided supporting evidence of altered polymer–filler interactions, consistent with DMA results showing a slight increase in glass T_g_ and reduced E’ in the rubbery region. Overall, pyrolysis transforms TSP into a more effective reinforcing filler by enhancing compatibility with the PLA matrix, particularly under flexural loading conditions.

The beneficial effect of biochar on the properties of FDM polymers can also be perceived by comparing biochar with other types of additives (Table 7). Biochar emerges as a sustainable alternative that uniquely balances mechanical reinforcement and crystallinity enhancements with trade-offs in thermal and printing performance. At low loadings (5–10 wt %), biochar can rival the stiffness improvements afforded by natural fibers while inducing a more moderate reduction in tensile strength compared to synthetic fibers and nanomaterials. Unlike inorganic fillers, which primarily stabilize dimensions under heat with minimal effects on polymer crystallinity, biochar simultaneously elevates PLA’s degree of crystallinity and causes a slight catalytic decrease in glass transition temperature, mirroring the behavior of carbon-based nanofillers but at a significantly lower cost and environmental burden. However, similar to fibrous and nanoscale additives, biochar requires careful dispersion to prevent agglomeration and an associated rise in extrusion torque and nozzle abrasion, challenges that can be addressed through optimized filament drying and extrusion parameters [67,68,69,70,71,72,73,74,75,76,77,78,79,80,81,82,83,84,85,86,87,88,89,90,91,92,93,94,95,96].

## 4. Conclusions

In this study, tomato stem waste was successfully converted into biochar and utilized as a filler in PLA composites for 3D printing. The research explored the effects of biochar on the mechanical and thermomechanical properties of the composites. The mechanical testing revealed that increasing biochar content in PLA composites significantly reduced tensile strength and elongation at break, particularly at 5% and 7.5% loadings, regardless of infill pattern. In contrast, flexural strength increased with biochar addition, while flexural modulus decreased, with both effects influenced by biochar content and infill geometry. Dynamic mechanical analysis revealed that the storage modulus in the glassy region was enhanced for composites with 5% biochar, indicating improved stiffness. However, a reduction in the storage modulus in the rubbery region for all biochar composites suggested potential changes in polymer-filler interfacial adhesion at higher temperatures. Thermal analysis indicated a slight increase in the glass transition temperature of PLA with the addition of biochar, pointing to restricted chain mobility. Based on the overall results, it appears that 5 wt.% is the optimum TSP-derived biochar concentration, between the two studied, to produce 3D-printed PLA/biochar composites.

These findings highlight that PLA/biochar composites offer a compelling route to industrial products ranging from lightweight automotive interior panels and consumer electronics enclosures to biodegradable packaging and water-filtration devices. The use of agricultural or forestry residues as biochar feedstock supports scalable filament production with minimal changes to existing extrusion and printing infrastructure. From an environmental standpoint, embedding stable carbon in printed parts not only valorizes waste biomass but also sequesters CO_2_ over the product life cycle, while reducing reliance on petroleum-derived fillers. Moreover, enhanced crystallinity and mechanical performance at low biochar loadings enable durable yet compostable components, aligning circular-economy principles with advanced manufacturing demands.

## Figures and Tables

**Table 1:** Summary of PLA/Biochar composite filaments.

Filament Type	Components		
	PLA	Biochar	Joncryl
	(wt.%)	(wt.%)	(wt.%)
Neat PLA	98	-	2
PLA-5% Biochar	93	5	2
PLA-7.5% Biochar	90.5	7.5	2

**Table 2:** Summary of PLA/biochar 3D-printed specimens.

Type of Test	Specimen Morphology	Infill Pattern	Number of Specimens		
			Neat PLA	PLA-5% Biochar	PLA-7.5% Biochar
Tensile Strength	ASTM D638 Standard, Type V	Concentric/ Rectilinear	5/5	5/5	5/5
Flexural Strength	rectangular parallelepiped 50 × 5 × 2 mm	Concentric/ Rectilinear	5/5	5/5	5/5
Dynamic Mechanical Analysis	rectangular parallelepiped 50 × 5 × 2 mm	Concentric/ Rectilinear	5/5	5/5	5/5

**Table 3:** Physicochemical parameters of tomato stem waste biochar.

Elemental Analysis		DLS		N_2_ Physisorption	
C (wt.%)	61.93	Particle diameter (μm)	2.2	BET surface area, S_BET_ (m^2^/g)	2.048
H (wt.%)	2.38	Hydrodynamic diameter (μm)	2.0		
N (wt.%)	2.21			Pore volume, V_P_ (cc/g)	0.015
O (wt.%)	33.48				

**Table 4:** TGA results of tomato stem waste biochar.

Temperature Range	*T*_onset_	*T_DTO max_*	*T*_end_	Mass Loss
(°C)	(°C)	(°C)	(°C)	(%)
24–220	24.3	81.6	132.4	4.65
220–520	403.6	472.8	514.3	9.18
520–760	637.0	684.0	752.8	9.86

Residual mass at 950 °C: 64.16%.

**Table 5:** Thermal transitions and crystallinity from DSC measurements.

Sample	1st Heating				2nd Heating			
	T_c_	T_cc_	T_m_	X_c_	T_c_	T_cc_	T_m_	X_c_
	(°C)	(°C)	(°C)	(%)	(°C)	(°C)	(°C)	(%)
Neat PLA	63	127	152	0.16	61.7	128	152.3	0
PLA-5% Biochar	66.7	121.7	150.7	0	62.7	125.7	151.4	0
PLA-7.5% Biochar	65.6	118.74	150.4	0	61.6	123.7	151.1	0

**Table 6:** Mechanical and thermomechanical properties of 3D-printed PLA/biochar composites.

Property	Neat PLA		PLA-5% Biochar		PLA-7.5% Biochar	
	Concentric	Rectilinear	Concentric	Rectilinear	Concentric	Rectilinear
**Tensile Testing**						
Tensile strength, σ_b_ (MPa)	41.5 ± 9.2	46.8 ± 12.8	32.9 ± 0.6	32.0 ± 0.2	33.4 ± 0.8	30.3 ± 0.8
Elongation at break, ε_b_ (%)	3.3 ± 0.3	4.6 ± 1.0	2.9 ± 0.1	2.9 ± 0.1	3.3 ± 0.1	3.0 ± 0.0
Young’s modulus, E_b_ (MPa)	1347 ± 155	1149 ± 175	1286 ± 2	1242 ± 8	1212 ± 19	1123 ± 19
**Flexural Testing**						
Flexural strength, σ_f_ (MPa)	52.3 ± 3.9	38.5 ± 2.6	72.9 ± 1.3	71.7 ± 4.0	68.9 ± 4.7	60.0 ± 3.7
Flexural strain at break, ε_f_ (%)	0.17 ± 0.03	0.13 ± 0.04	0.17 ± 0.01	0.20 ± 0.02	0.20 ± 0.03	0.20 ± 0.07
Modulus of elasticity, E_f_ (MPa)	984.0 ± 59.3	657.8 ± 56.7	772.2 ± 223.3	540.1 ± 69.7	520.7 ± 31.4	492.2 ± 66.4
**Dynamic Mechanical Analysis**						
Storage modulus, E’, at 30 °C (MPa)	1298	1228	1385	1310	1183	1145
Storage modulus, E’, at 65 °C (MPa)	147	148	95	119	117	104
Glass transition temperature, T*_g_* (°C)	59.3	59.8	59.5	60.4	57.4	59.2

**Table 7:** Effect of biochar addition on the properties of 3D-printed PLA composites compared to other types of additives.

Additive Type	Typical Additives	Mechanical Impact	Thermal Stability	Printability
Biochar [21,61,69,70,91]	Biochar from different sources at 5–40 wt.% loadings	Mechanical reinforcement at low (5–10 wt.%) loadings; mechanical properties decrease in higher (>10 wt.%) loadings	Minor changes in thermal stability, glass transition temperature, and degree of crystallinity	Potential increase in agglomeration, extrusion torque, and nozzle abrasion; moisture sensitivity
Natural fibers [42,76,79,83,87,89]	Wood powder, natural fibers, etc.	Moderate increase in stiffness; reduced tensile strength	Minor improvement in heat deflection	Increased nozzle wear; moisture sensitivity
Synthetic fibers [73,74,77,78,80,84,90,92]	Carbon fibers, glass fibers, Kevlar, etc.	Significant gain in tensile strength and stiffness	Substantial increase in glass transition	Higher extrusion torque; nozzle clogging risk
Inorganic particles [67,75,81,82,88,94,95,96]	Calcium carbonate, talc, metal oxides, etc.	Improved dimensional stability; slight stiffness gain	Enhanced dimensional accuracy	Better melt flow; reduced shrinkage
Nanomaterials [68,71,72,85,86,93]	Graphene, carbon nanotubes, etc.	Exceptional rise in strength and modulus	Considerable increase in thermal stability	Requires controlled dispersion; potential nozzle abrasion

## Data Availability

The original contributions presented in this study are included in the article. Further inquiries can be directed to the corresponding authors.

## Abbreviations

The following abbreviations are used in this manuscript:

3D	Three dimensional
AM	Additive Manufacturing
ANOVA	Analysis of Variance
ASTM	American Society for Testing and Materials
BET	Brunauer–Emmett–Teller
DLS	Dynamic Light Scattering
DMA	Dynamic Mechanical Analysis
DSC	Differential Scanning Calorimetry
FDM	Fused Deposition Modeling
FFF	Fused Filament Fabrication
PBS	Poly(Butylene Succinate)
PLA	Poly(Lactic Acid)
PSD	Particle Size Distribution
SEM	Scanning Electron Microscopy
SLS	Selective Laser Sintering
TGA	Thermal Gravimetric Analysis
TSP	Tomato Stem Powder
UV	Ultraviolet
